# A proteomic and RNA-seq transcriptomic dataset of capsaicin-aggravated mouse chronic colitis model

**DOI:** 10.1038/s41597-022-01637-3

**Published:** 2022-09-07

**Authors:** Kexin Chen, Silan Shen, Yiding Chen, Mingshan Jiang, Kehan Hu, Yuheng Zou, Lili Li, Zhen Zeng, Chunxiang Ma, Yuan Dang, Hu Zhang

**Affiliations:** 1 Department of Gastroenterology, West China Hospital, Sichuan University, Chengdu, China; 2 Centre for Inflammatory Bowel Disease, West China Hospital, Sichuan University, Chengdu, China; 3 Laboratory of Inflammatory Bowel Disease, Institute of Immunology and Inflammation, Frontiers Science Center for Disease-Related Molecular Network, West China Hospital, Sichuan University, Chengdu, China

**Keywords:** Inflammatory bowel disease, Immunological disorders

## Abstract

An inappropriate diet is a risk factor for inflammatory bowel disease (IBD). It is established that the consumption of spicy food containing capsaicin is strongly associated with the recurrence and worsening of IBD symptoms. Moreover, capsaicin can induce neutrophil accumulation in the lamina propria, contributing to disease deterioration. To uncover the potential signaling pathway involved in capsaicin-induced relapse and the effects of capsaicin on neutrophil activation, we performed proteomic analyses of intestinal tissues from chronic colitis mice following capsaicin administration and transcriptomic analyses of dHL-60 cells after capsaicin stimulation. Collectively, these multiomic analyses identified proteins and genes that may be involved in disease flares, thereby providing new insights for future research.

## Abstract (summary)

**Table:** 

Measurement(s)	protein expression profile • gene expression profile
Technology Type(s)	mass spectrametry • RNA sequencing
Factor Type(s)	time, experimental factor (capsaicin)
Sample Characteristic - Organism	Mus musculus • Homo sapiens

## Background & Summary

Inflammatory bowel disease (IBD), comprising ulcerative colitis (UC) and Crohn’s disease (CD), is a lifelong and incurable disease that provokes a relapsing-remitting course in most patients^1^. In recent decades, the incidence of IBD has rapidly risen in newly industrialized countries due to environmental changes^2^. This refractory and cost-consuming disease has increased the burden of the disease on patients and society^3–5^. Some studies have suggested that an inappropriate diet, as one environmental factor, plays a critical role in the onset, recurrence, and progression of IBD^6–9^. Specifically, spicy food is found to be closely related to the recurrence of the disease. For example, a questionnaire related to dietary intake and IBD symptoms revealed that over 80% of IBD patients perceived spicy food as a risk factor for relapse^10^. A large online questionnaire cohort study reported that spicy food intake can worsen symptoms^11^. However, to date, there are so far not enough proteomic profiling data to explain the mechanisms of diet-induced disease flares. Such a lack of convincing biological evidence leaves this topic controversial. In this study, our main aim was to reveal the potential signaling pathways related to disease activity induced by spicy food through proteomic analyses of colon tissues from mouse models of DSS-induced chronic colitis treated with capsaicin.

Proteomics, which is widely utilized to identify differential protein expression, mainly relies on mass spectrometry (MS) technology^12^. Data-dependent acquisition (DDA) and data-independent acquisition (DIA) scan modes are used to acquire the MS data. The DDA model uses a narrow m/z window to scan target ions and reduces the percentage of interfering ions, which can provide some high-quality debris information^13^. In our study, we used the DDA model for peptide quantification combined with the front-end high-field asymmetric waveform ion mobility spectrometry (FAIMS) interface. FAIMS installed between the MS vacuum chamber and ion source can reduce chemical noise and matrix interference to improve detection durability and sensitivity by compensating voltage for the electrode^14–17^.

Capsaicin is derived from chili peppers and bestows their characteristic spicy flavor^18^. Its receptor is the transient receptor potential vanilloid 1 (TRPV1), which is expressed in a wide variety of cells, including dorsal root ganglion (DRG) neurons^19^, epithelial cells^20,21^, neutrophils^22^, dendritic cells (DCs), and macrophages^23^. The widespread expression of TRPV1 in immune cells indicates that it plays a role in immune regulation. Studies have shown that Ca2+ influx through TRPV1 in neutrophils can lead to the release of pro-inflammatory factors^22^. Similarly, the activation of TRPV1 has been shown to induce neutrophil accumulation and thus exacerbate experimental colitis^24,25^. These findings highlight the critical functions of TRPV1 in neutrophils. However, the effect of neutrophils/TRPV1 on IBD is complex^26–29^ and additional research is required to properly characterize their positive and negative impact on disease. High-throughput RNA sequencing (RNA-seq) is widely used for total RNA expression profiling. Neutrophils have a short lifespan, but they share many key characteristics with differentiated HL-60 neutrophil-like cells (dHL-60)^30^. Thus, dHL-60 cells are frequently used in neutrophil research. To identify the characteristics of neutrophils treated with capsaicin, we performed RNA-seq of capsaicin-processed differentiated HL-60 cells.

**Fig. 1:**
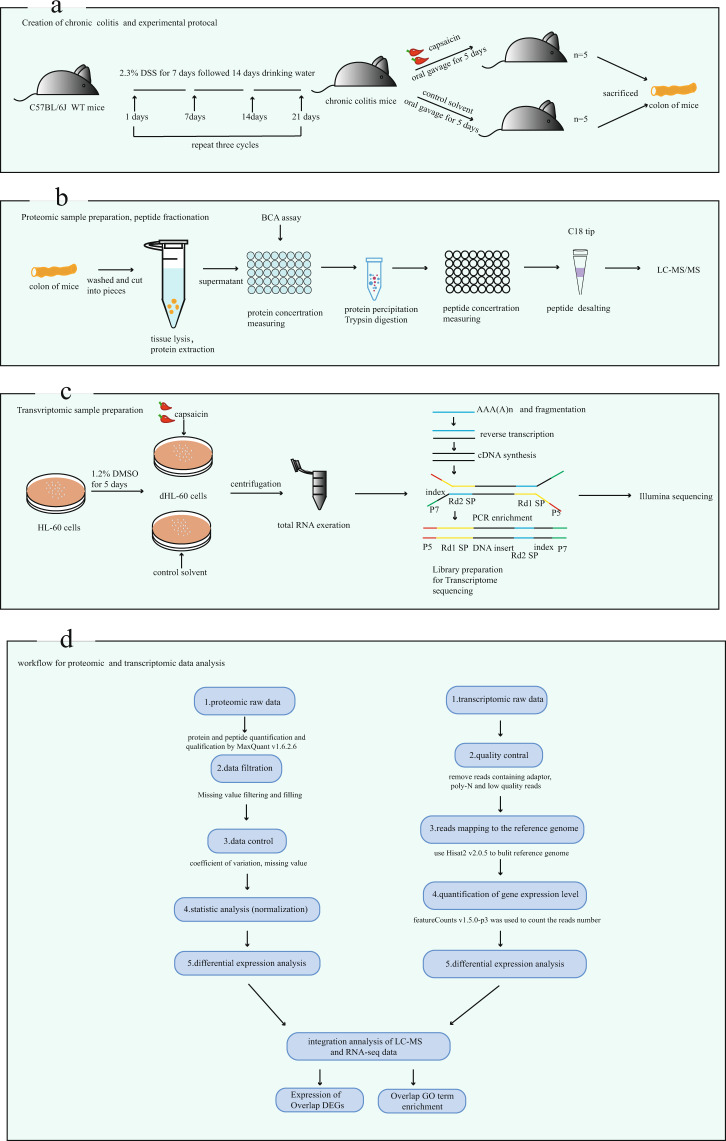
Workflow of sample preparation and data analysis. (**a**) The establishment of chronic colitis mice model and the progress of the experiment. Two groups of chronic colitis mice were established (n = 5). (**b**) The progress of proteomic sample preparation for LC‒MS. In total, 10 samples from two groups (CAP; DSS) were used. (**c**) Transcriptomic sample preparation and the process of detection. (**d**) The flow chart of the proteomic and transcriptomic data analysis.

In this study, we collected colon tissues from capsaicin oval-gavage-treated mice of chronic colitis for proteomic data acquisition. We identified differentially expressed proteins that were closely associated with inflammatory pathways. We also generated capsaicin-stimulated neutrophil-like cells, dHL-60, to build transcriptomic maps of their gene expression patterns (Fig. 1). In general, this study identifies the key pathways involved in the recurrence of capsaicin-aggravated colitis and the effects of neutrophils on intestinal inflammation. As such, our study helps elucidate the underlying mechanisms of IBD.

## Methods

### Cell culture

**Fig. 2:**
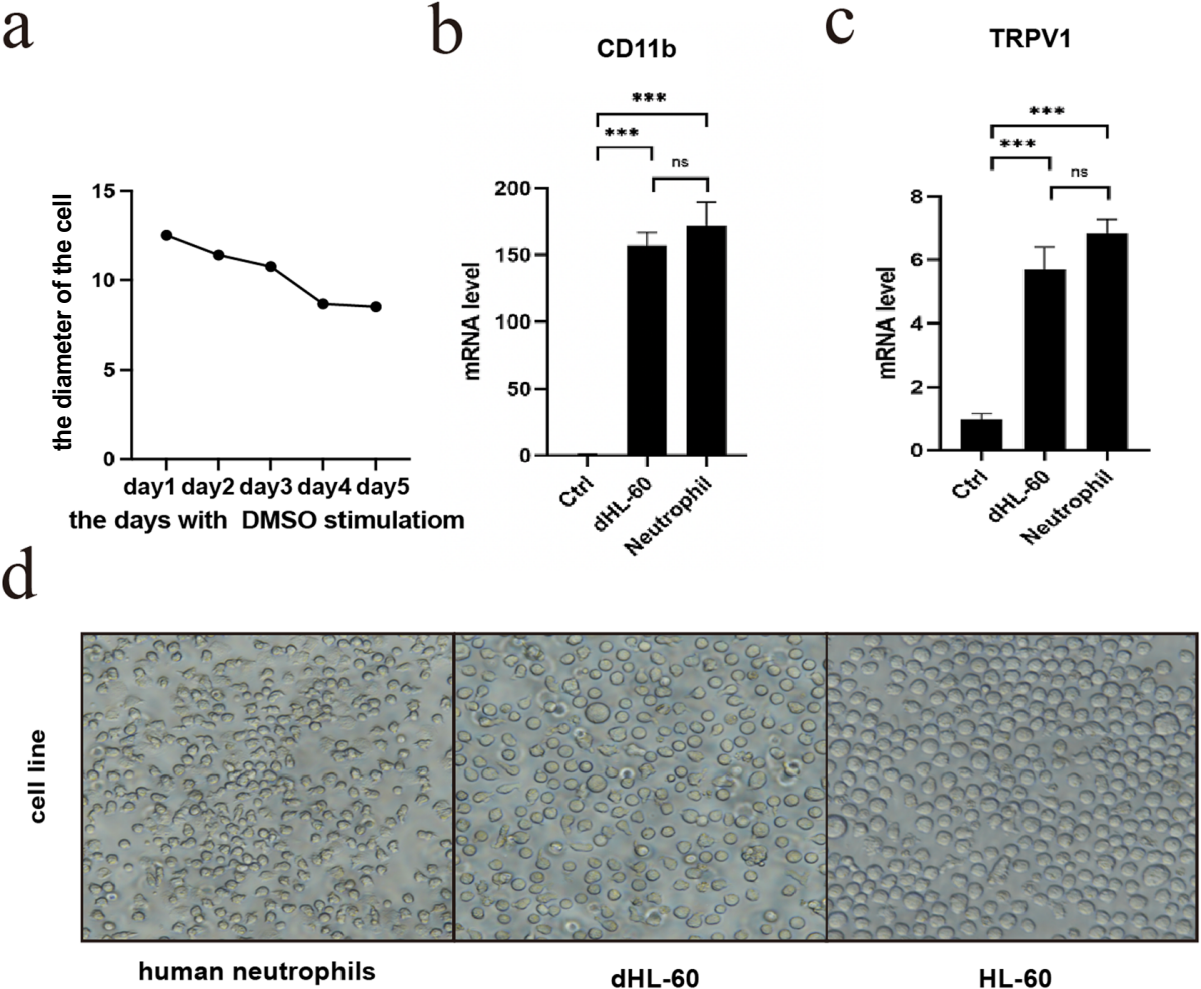
Differentiation assessment of the dHL-60. (**a**) The diameter changes of the cells after induction. (**b**) The expression profile of CD11b. (**c**) The alteration of TRPV1 expression after induction. (**d**) Comparison of the cell lines. Photomicrograph (20X) of cultured cells. All values represent the mean with SE; a two-sided t test. *P < 0.05, **P < 0.01, ***P < 0.005.

Differentiated HL-60 neutrophil-like cells (dHL-60) were induced by stimulation with 1.2% dimethyl sulfoxide (DMSO) over 5 days from the human leukemic cell line HL-60 (ATCC CCL-240). HL-60 and dHL-60 cells were maintained in RPMI-1640 medium supplemented with 10% serum, which was changed once every three days. Capsaicin (Sigma‒Aldrich) was reconstituted in DMSO at a final concentration of 400 mM and then added to the medium. As described previously^30^, we used three criteria to assess the effectiveness of HL-60 differentiation: i) the cell diameter was smaller after induction (Fig. 2a,d); ii) CD11b, as a biomarker of neutrophils, was increased (Fig. 2b); and iii) TRPV1 which is the receptor of capsaicin was dramatically increased at the RNA expression level and was equal to the expression of human peripheral neutrophils (Fig. 2c).

### Preparation of colonic tissue from chronic colitis mice

Wild-type C57BL/6 J male mice (8 weeks old) were obtained from the Chengdu Dossy Experimental Animals Company (Chengdu, China) and maintained in the animal facilities at the Frontier Medical Center of Chengdu with automatically controlled temperature and humidity. A chronic colitis model was induced by administering 2.3% dextran sulfate sodium (DSS) (molecular mass, 36 000–50 000, MP Biomedicals, Solon, Ohio, USA) in autoclaved tap water over 7 days and changed to distilled water for the next 14 days. Then the 21-day cycle (7 days DSS solution and 14 days distilled water) was repeated twice more for a total of three cycles^31^. The establishment of colitis was judged based on body weight, stool characteristics, and hematochezia. After three induction periods, the mice were provided with capsaicin (0.20 mg/kg/d) or vehicle control (10% Tween 80–10% ethanol-80% NS) via oral gavage over 5 consecutive days. Then, the mice were sacrificed by cervical dislocation. The colon, from the ileum to the anus, was washed with cold phosphate-buffered saline (PBS). Colon tissue was then deposited in liquid nitrogen for proteomics (Fig. 1a). All animal procedures received ethics approval from the Institutional Animal Care and Use Committee of West China Hospital.

### Sample preparation for proteomics

The process of proteomic sample preparation is summarized in Fig. 1b. As described previously, the colonic tissues were isolated from mice with chronic colitis after treatment and were frequently frozen in liquid nitrogen. Ten milligrams of colonic tissue was added to 1 ml of T-PER Lysis Buffer (Thermo Fisher Scientific) and was supplemented with a complete Protease Inhibitor Cocktail (Roche, 4693132001). Then, the samples were lysed with an ultrasonic tissue homogenizer (60 HZ × 1 min), incubated at 4 °C for 10 minutes, homogenized again (60 HZ × 30 sec), and incubated for 30 minutes. Lysates were centrifuged at 16,000 × g for 20 minutes at 4 °C and the supernatant was retained. Then, the protein concentration was determined using a BCA assay kit (Thermo Fisher Scientific) according to the manufacturer’s protocol. For each sample, 300 μg of protein sample was diluted with 50 mM NH_4_HCO_3_ (Sigma) to 100 μl and 500 μl of prechilled acetone (Thermo Fisher Scientific) was added and stored overnight at −20 °C. The sediment was washed with 500 μl prechilled acetone and centrifuged at 15,000 × g for 5 minutes at 4 °C. Then, we removed the supernatant and dissolved the sediment with 50 μl 8 M urea for 2 hours, added 1 μl 1 M DTT (Sigma), and incubated the mixture at 30 °C for 2 hours. After that, 2.5 μl of 1 M IAM was added and the samples were placed in the dark for 30 minutes. Next, the samples were diluted to 200 μl with 8 M urea, added to the PALL10K filtration capsule (Millipore), and centrifuged at 10,000 g for 15 minutes. Urea (8 M) and 50 mM NH_4_HCO_3_ were used to wash the protein sample. Then, the protein was resuspended in 100 μl of 50 mM NH_4_HCO_3_, and 6 μg of sequencing grade modified trypsin (Sigma) was added and digested at 37 °C for 16 hours and then centrifuged at 10,000 × g for 15 minutes. Fifty microliters of 50 mM NH_4_HCO_3_ was used to wash the peptide twice, and the effluents were combined with TFA (0.4%) to end the reaction. The peptide concentration was quantified by the Pierce Quantitative Colorimetric Peptide Assay Kit (Thermo Fisher Scientific). Finally, the peptides were desalted using Pierce C18 Tips (Thermo Fisher Scientific), and the tips were washed using 10 μl 50% ACN/H2O and 0.1% TFA/H2O. Subsequently, the peptide was eluted with 0.1% TFA (Thermo Fisher Scientific) and 50% ACN (Thermo Fisher Scientific) and then freeze-dried for MS analysis. The peptide concentration was measured by a peptide assay kit (Thermo Fisher Scientific) (Table 1).

**Table 1:** Peptide concentration before the mass spectrometry.

samples	CAP_1	CAP_2	CAP_3	CAP_4	CAP_5	DSS_1	DSS_2	DSS_3	DSS_4	DSS_5
peptide concentration (ng/μl)	186.3158	168.6948	157.8947	127.3684	176.8421	409.4737	311.5789	189.4737	314.7368	327.3684

### DDA-MS data acquisition

All samples were resolved in 10 µL of mobile phase A solvent (0.1% formic acid [v/v]) to be analyzed on the Orbitrap Exploris 480 mass spectrometer (ThermoFisher Scientific, USA). Approximately 1 μg of the peptide was isolated from each sample on the EASY-nLC 1200 system (ThermoFisher Scientific, USA) using an in-house packed 25 cm, 75 μm i.d. capillary column with 1.9 μm Reprosil-Pur C18 resin (Dr. Maisch, Ammerbuch, Germany) at flow rates of 300 nL/minutes over 60 minutes linear gradient. In DDA-MS, data were acquired on an Exploris 480 mass spectrometer with FAIMS running dual compensation voltages at −45 V and −65 V and using EASY-IC. For MS1, the full scan orbitrap resolution was set to 60,000, the scan was set to 350–1500 (m/z) and the full scan normalized AGC target was 300% with a maximum injection time of 50 ms. For MS2, the full scan orbitrap resolution was set to 15,000 with a 75% normalized AGC target. The maximum injection time was set at 22 ms. The precursor intensity threshold was kept at 5e4. The isolation window was 1.6 m/z with 30% normalized collision energy^17^.

### Protein identification and quantification

DDA raw data files were processed by using Proteome Discoverer 2.4 software with the standard settings as described previously^32^. Briefly, the MS/MS spectra were searched against the UniProt mouse database (downloaded in April 2020) using a label-free quantification method with trypsin as a protease and allowing up to two missed cleavages. Alkylation of cysteines was used as fixed modification, and oxidation of methionine and N-terminal acetylation were used as variable modifications. The false discovery rate (FDR) for protein identification was set to 0.01. The finally identified proteins were used for subsequent analyses.

### Transcriptomic sample preparation

#### RNA extraction

The treated cells were counted and collected after washing with PBS buffer. Total RNA was isolated from cells with TRIzol (Ambion, USA) according to the manufacturer’s instructions. In short, chloroform was added as one-fifth of the total volume, shaken vigorously for 15 seconds, rested for 3 minutes, and centrifuged at 12,000 rpm for 15 minutes at 4 °C. Then the water phase was moved to a new centrifuge tube with an equal volume of isopropyl alcohol and inverted to mix and incubated for 10 minutes. This was centrifuged, the supernatant was discarded, and then 1 ml 75% alcohol diluted with RNase-free water was added. Agarose gel electrophoresis was used to analyze the integrity of RNA and detect the existence of DNA contamination. An Agilent 2100 Bioanalyzer 2100 was used to detect RNA integrity precisely. RNA purity was assessed by the ratio of OD260/280 and OD260/230.

### Library construction for transcriptomic analyses

Total RNA over 1 μg was used as the initial RNA for library preparation. The kit used for library preparation was the NEBNext® UltraTM RNA Library Prep Kit for Illumina® (NEB, USA). First, mRNA with a polyA tail was enriched by OligO (dT) magnetic beads and then interrupted randomly in NEB Fragmentation Buffer by divalent cations. Then the interrupted mRNA was set as a template and random oligonucleotides were used as primers to synthesize the first cDNA strand under the M-MuLV reverse transcriptase system. The RNA strand was cleared by RNaseH. Under the DNA polymerase I system, the second cDNA strand was synthesized. Subsequently, the purified double-stranded cDNA was processed, and cDNA of 250–300 bp was purified by AMPure XP beads. After amplification, the PCR products were purified again using AMPure XP beads. Finally, the library was prepared. The preliminary quantification was conducted by Qubit 2.0 Fluorometer and diluted to 1.5 ng/μl. Precise quantification was performed by qRT‒PCR to assess the library.

After library pooling, Illumina novaseq6000 was used for sequencing and generating a 150 bp paired terminal reading. In the sequencing flow cell, the four types of dNTPs with fluorescent, DNA polymerase, and primer were added for amplification. When every detection cluster extended the complementary chain, the dNTP labeled by fluorescence was detectable so that the sequencing information could be captured by the sequencer (Fig. 1c).

## Data Records

All proteomic data described in this study have been stored in the Proteome change Consortium via PRIDE https://identifiers.org/pride.project:PXD032186 with the accession number PXD032186^33^. The RNA-seq data are available at the NCBI Gene Expression Omnibus (GEO) with dataset identifier GSE198304^34^. These data contained raw read count data of RNA-seq, gene counts, and FPKM values for all samples. Tables 2 and 3 provide the sample group information, treatment, replications, and accession number of the datasets.

**Table 2:** Proteomics datasets stored in PRIDE.

Group	Tissue	Treatment	Replicates	Method	Data collection	Data
DSS	Colon	DSS and control solvent	5X	Protein extraction	Mass spectrometry	PXD032186
CAP	Colon	DSS and capsaicin	5X	Protein extraction	Mass spectrometry	PXD032186

**Table 3:** RNA-seq datasets stored in GEO.

Group	Treatment	Replicates	Method	Data collection	Data
CTRL	Control solvent	3X	RNA extraction	RNA-seq	GSE198304
CAP_8H	Capsaicin for 8 hours	3X	RNA extraction	RNA-seq	GSE198304
CAP_16H	Capsaicin for 16 hours	3X	RNA extraction	RNA-seq	GSE198304
CAP_24H	Capsaicin for 24 hours	3X	RNA extraction	RNA-seq	GSE198304

## Technical Validation

### RNA-seq quality control

**Fig. 3:**
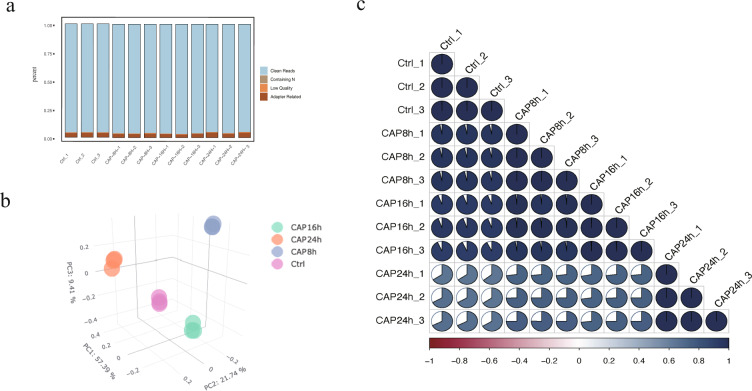
Quality control of transcriptomic (**a**) Classification of raw reads: clean reads, containing N, low quality, adapter related. (**b**) A three-dimensional principal component analysis (PCA) plot was performed to assess the variability of samples within and between groups. The plot of the first three axes from a PCA based on counts of the gene across all samples control (red, n = 3) and CAP8H (blue, n = 3); CAP16H (green, n = 3) and CAP24H (orange, n = 3). (**c**) The correlation of the duplicated samples.

**Fig. 4:**
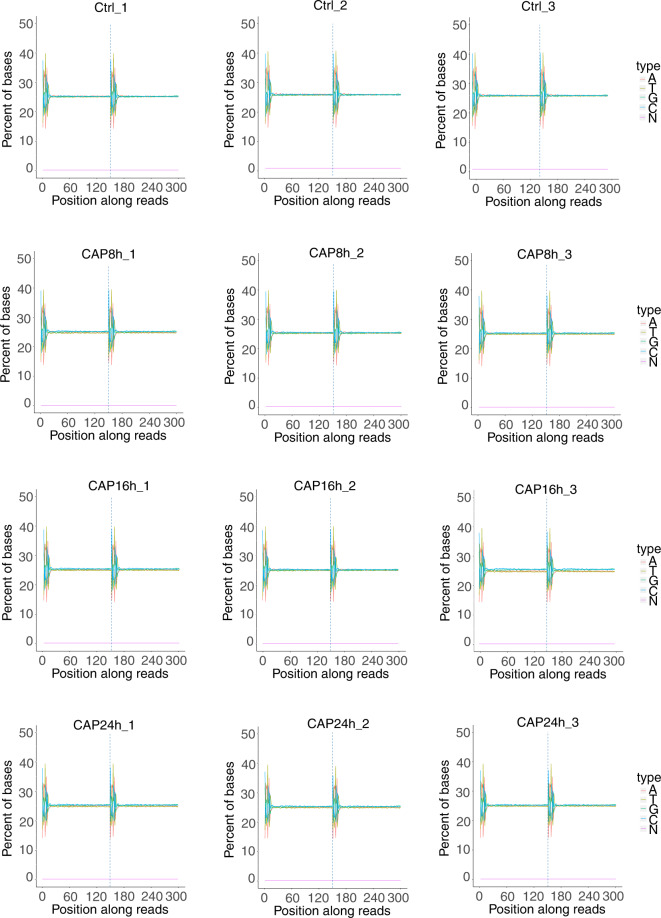
Distribution of the proportions of guanine (G) and cytosine (C) in a nucleotide sequence (GC contents) in transcriptomic samples. The GC contents in the four groups are shown. Horizontal coordinates of the graph are the base position of the reads, and the vertical coordinates are the percentages of the five base types of ATGCN.

Quality control of all samples was performed using Fastp. The raw data of the sequencing data included adaptor sequencing and low-quality reads. To guarantee the quality of the sequencing analysis, the raw data required filtration. Reads with an adaptor, reads with N (N means base information cannot be determined), and low-quality base numbers with low Phred scores (less than or equal to 20) more than 50% of the whole reads were removed. The component proportion diagram of the filtered sequencing data for each group is shown in Fig. 3a. The Q20, Q30, and GC percent were calculated (Table 4), and the GC contents of the experimental group and control groups are shown in Fig. 4, while the remaining samples all had similar GC content distributions. Principal component analysis (PCA) was used to assess the similarities within samples and whether the samples could be grouped well (Fig. 3b). Error rate statistics were calculated, and the error rate of samples without contamination and specific processes was found to be less than 0.03% (Table 4). An index of the reference genome was built using HISAT2v2.0.5, which can produce a database of splice junctions. The paired terminal clean reads were mapped to the reference genome using the genome reference document. A total of 37 million to 44 million reads were mapped to the reference genome, where more than 80% of the reads were uniquely mapped (Table 4). The distribution of the mapping genome district is shown in Table 5. Finally, to assess the correlation of samples from the same group, we used Pearson’s test to calculate the correlation coefficient. The samples in one experimental group exhibited a robust similarity (Fig. 3c). After quality control, a total of 9,234 genes were identified in the four groups.

**Table 4:** Quality of the transcriptomic data.

Samples	Insert mean	Raw reads	Clean reads	Unique map	Error rate	Q20	Q30	GC_pct
CTRL_1	270.263015	46393688	45366372	40807528	0.02	98.27	94.87	50.08
				89.95%				
CTRL_2	276.253272	40076918	39167786	35317655	0.02	98.33	95.02	50.03
				90.17%				
CTRL_3	279.501619	41948558	41015416	37030522	0.02	98.35	95.06	50.11
				90.28%				
CAP8h_1	280.42114	45205394	44327580	39953545	0.02	98.25	94.82	50.38
				90.13%				
CAP8h_2	284.804996	46174918	45312474	40937899	0.02	98.36	95.07	50.14
				90.35%				
CAP8h_3	282.903598	43320518	42410542	38299239	0.02	98.32	95	50.31
				90.31%				
CAP16h_1	280.317545	45940974	45097508	40619275	0.02	98.42	95.23	50.39
				90.07%				
CAP16h_2	281.97131	45282430	44538370	40167480	0.02	98.43	95.26	50.16
				90.19%				
CAP16h_3	281.448836	46722614	45806726	41420280	0.02	98.39	95.14	50.67
				90.42%				
CAP24h_1	269.580855	46783774	45603100	41202102	0.02	98.44	95.3	50.47
				90.35%				
CAP24h_2	273.931073	43048290	42179844	38102712	0.02	98.36	95.08	50.38
				90.33%				
CAP24h_3	274.084105	42275524	41189068	37155656	0.02	98.25	94.82	50.33
				90.21%

**Table 5:** Sample comparison area.

Sample	Exon	Intron	Intergenic
Ctrl_1	5382168796(82.4146%)	828085462(12.6801%)	320346390(4.9053%)
Ctrl_2	4661408617(82.5985%)	709450038(12.5712%)	272593654(4.8303%)
Ctrl_3	4862478252(82.2548%)	761475087(12.8813%)	287526941(4.8639%)
CAP8h_1	5220184212(81.8008%)	866291011(13.5749%)	295106693(4.6244%)
CAP8h_2	5371981596(82.2278%)	865832863(13.2531%)	295236105(4.5191%)
CAP8h_3	4966172441(81.2915%)	861115993(14.0956%)	281809659(4.613%)
CAP16h_1	5439957641(83.6688%)	754567473(11.6056%)	307250798(4.7256%)
CAP16h_2	5429628434(84.5426%)	695405602(10.8279%)	297322256(4.6295%)
CAP16h_3	5592457562(84.5483%)	721375106(10.9059%)	300681170(4.5458%)
CAP24h_1	5651165273(86.0521%)	633308980(9.6436%)	282674335(4.3044%)
CAP24h_2	5155626144(84.9197%)	652907375(10.7542%)	262640714(4.326%)
CAP24h_3	4954761082(83.7145%)	696843982(11.7737%)	267036292(4.5118%)

### Transcript expression profiling and differential expression analysis

**Fig. 5:**
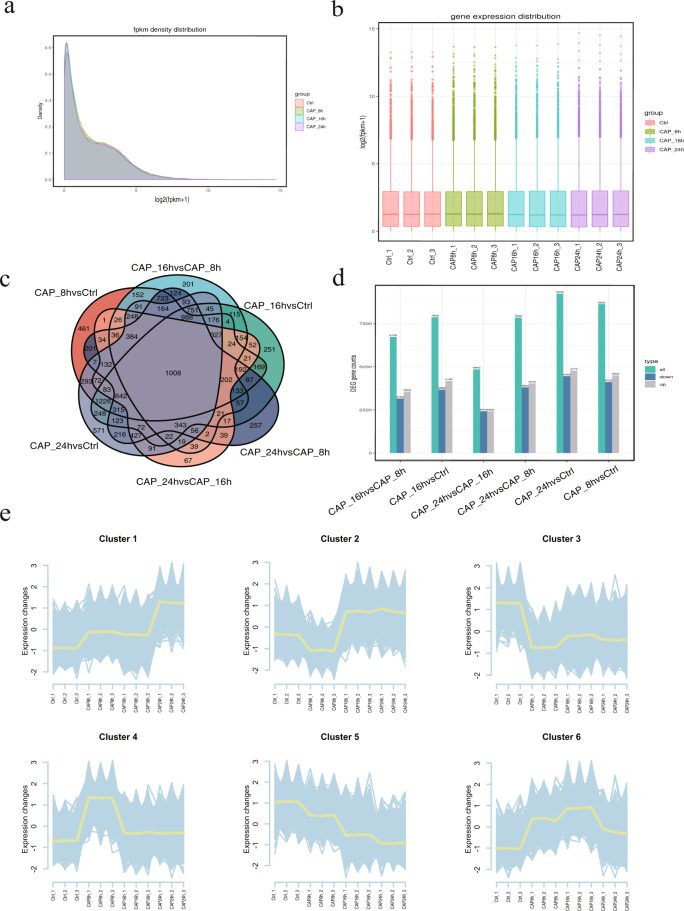
Differential expression gene analysis. (**a,b**) FPKM density distribution of the sequencing data. The X-axis represents the log2(FPKM + 1) value of the genes, and the Y-axis represents the distribution density of the genes with corresponding expression levels. (**c**) Venn diagram of the differentially expressed genes in comparation groups. (**d**) Summary of up- and down-regulated genes in different comparison groups. (**e**) Cluster line diagram of differentially expressed genes.

FeatureCounts (1.5.0-p3) was used to calculate the mapping reads of each gene. Then according to the length of the genes, we calculated the Fragments Per Kilobase of transcript sequence per Millions base pairs sequenced (FPKM) and the mapping reads of each gene. A summary of the FPKM of RNA-seq is shown in Fig. 5a,b. The DESeq2 R package (1.16.1) was used to test for differential gene expression in the comparator groups (three biological replicates were used per group). To control for the false discovery rate, the adjusted *p* value was calculated with the Benjamini‒Hochberg method^35^. The thresholds for a differential expression were adjusted as *p* value < 0.05 and log2|fold change| > 0. To reveal the unique and common differentially expressed genes in comparison groups, we generated Venn diagrams and identified 1,008 genes that were common and differentially expressed across the six comparison groups (Fig. 5c). The detailed expression profiles in six comparison groups are shown in Fig. 5d. Finally, we performed the clustering of differentially expressed genes by using the gene cluster tread tool in Hiplot (https://hiplot.org), an online web service for biomedical data visualization. Differentially expressed genes (DEGs) were divided into several clusters, and the genes in the same cluster had similar expression trends in different groups (Fig. 5e).

### Proteomic quality control and imputation

**Fig. 6:**
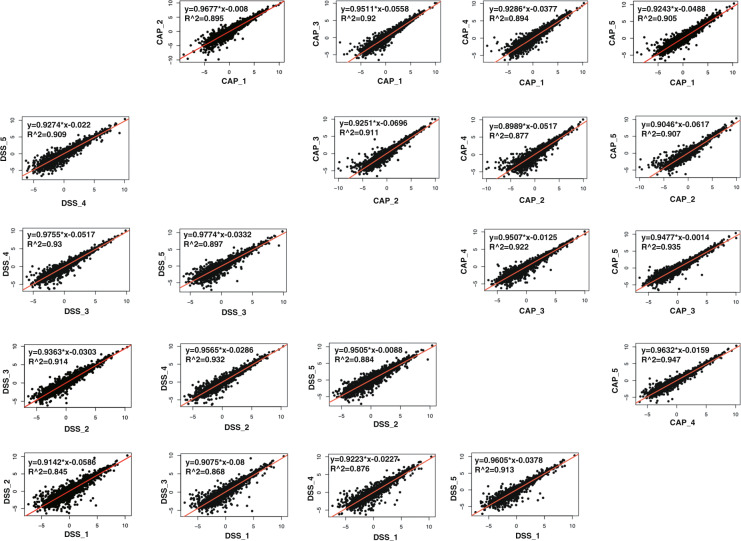
Comparison of quantitative information between biological replicates in the CAP group (capsaicin treatment) and DSS groups (vehicle control treatment). Each group had five sample replicates, and every two samples were compared.

**Fig. 7:**
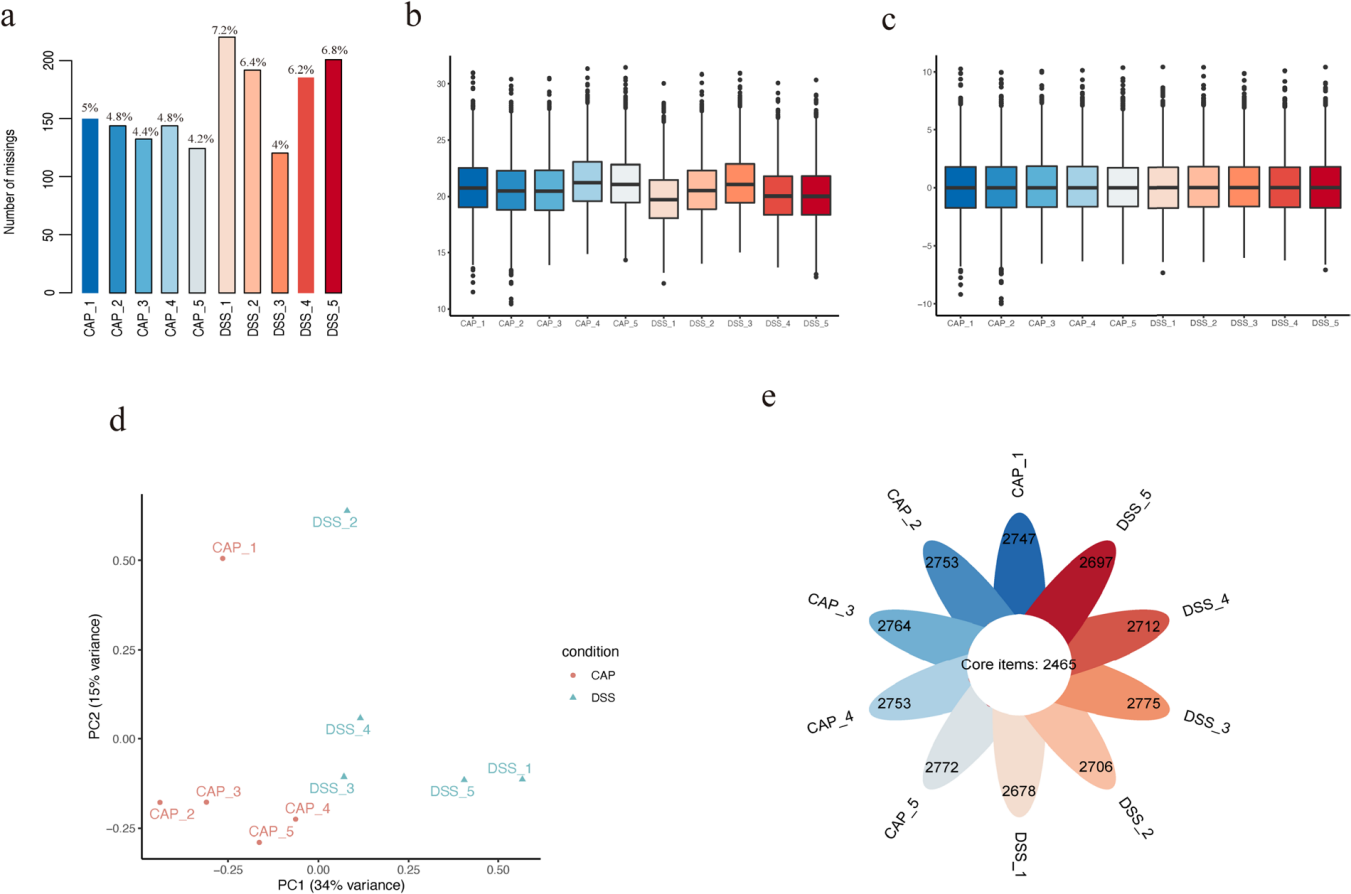
Quality control of proteomics. (**a**) Percent and count of the missing value in each sample. (**b**) Distribution of the quantitative data before missing value imputation within samples. The Y-axis represents the logarithmically transferred quantitative value. (**c**) Distribution of the missing value imputed proteomic data after normalization and logarithmic transformation. (**d**) Principal component analysis (PCA) score plot shows the discrepancies between groups. (**e**) The count of common items within samples.

First, to assess whether the biological replicates in the same group were quantitatively similar, we calculated the correlation coefficient for two samples in each group and found that the between-sample reproducibility was good (Fig. 6). Second, the missing value of each sample was evaluated and the high proportion of missing values was filtered. Then, 2,763 distinct proteins were retained with an average missing rate of 5.38%, as shown in Fig. 7a. The data distribution after the logarithmic transformation is shown in Fig. 7b. Third, imputation of missing values was performed by using NAguideR as previously described^36^. To minimize errors derived from the system and samples caused by processing, loading, and preclassification, normalization and log2 transformation were performed using an online platform (https://www.omicsolution.com/wkomics/main/). The distribution of processed data is shown in the boxplot of Fig. 7c. We also performed three-dimensional PCA, which showed good separation between the capsaicin (CAP) groups and control (DSS) groups (Fig. 7d). Figure 7e illustrates the overlapping proteins within the two experimental groups, and most of the identified proteins were common in a total of ten samples.

### Identification of differentially expressed proteins

Data processing for differential expression analysis was performed as follows: (i) missing value filtration and imputation; (ii) logarithmic transformation; and (iii) data normalization. Then, the differentially expressed proteins were identified by the Limma R package by calculating the adjusted *p* value and fold change value^37^. First, the group list was built, and a linear model fit was performed. Then, the *p* value of the protein was calculated by the empirical Bayes test. Adjusted *p* values were calculated by the Bonferroni‒Holm method to control for multiple comparisons. We determined differential protein expression based on a fold change > 0 and an adjusted *p* value < 0.05. More than 50 proteins were determined to be differentially expressed.

### Integrative analysis of proteomic and transcriptomic data

**Fig. 8:**
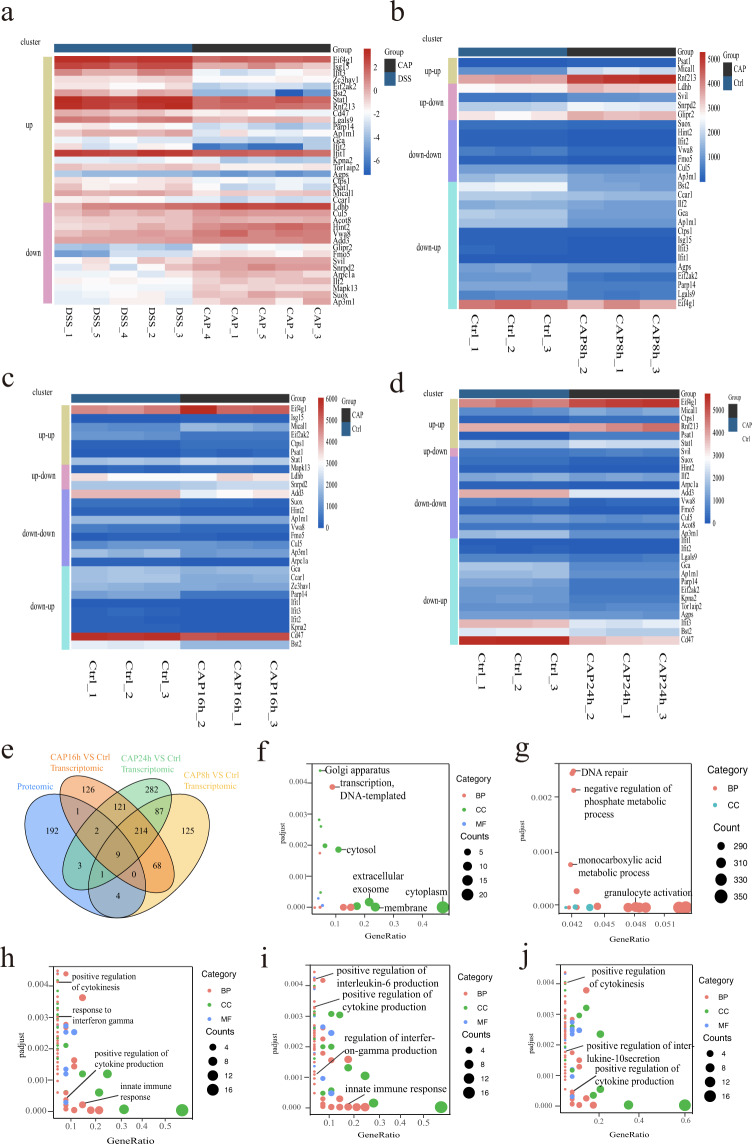
Overlapping genes profiles and GO enrichment. (**a**) Expression profile of overlapping genes at the protein level. (**b**) Overlapping genes expression profile in the CAP_8 h vs Ctrl group. (**c**) Overlapping genes expression profile in the CAP_16 h vs Ctrl group. (**d**) Overlapping genes expression profile in the CAP_24 h vs Ctrl group. (**e**) Venn diagram of overlapping GO term enrichment of DEGs and DEPs. (**f**) GO term enrichment of DEPs. (**j**) GO term enrichment of DEGs. (**h**) GO term enrichment of overlapping genes in the CAP_8 h vs Ctrl group. (**i**) GO term enrichment of overlapping genes in the CAP_16 h vs Ctrl group. (**j**) GO term enrichment of overlapping genes in the CAP_24 h vs Ctrl group.

In this study, 2,763 proteins were identified by mass spectrometry and 9,234 genes were detected by RNA sequencing. We used three strategies to accomplish integrative analysis. First, based on proteomic data, more than 50 proteins were differentially expressed. DEGs in transcriptomic data were compared with this list to find the overlap. Figure 8a shows the expression profile of overlapping genes at their protein level. Second, according to the correspondences and discrepancies between RNA and protein expression, we classified overlapping genes into four clusters: (i) up-up (increased at both the RNA and protein level), (ii) up-down (increased at the RNA level, and decreased at the protein level), (iii) down-down (decreased at both the RNA level and protein level), (iv) down-up (decreased at the RNA level and increased at the protein level) (Fig. 8b–d). Third, DEPs and DEGs at their representative GO term enrichment also showed coverage (Fig. 8e). In the top 20 GO terms enrichment of DEPs, the cellular component was dominant (Fig. 8f), while in DEGs, the biological process was prominent (Fig. 8g). For overlapping genes, GO enrichment analysis revealed that enriched pathways highly related to the immune response and inflammatory factor regulation in the CAP_8 h group (Fig. 8h), CAP_16 h group (Fig. 8i), and CAP_24 h group (Fig. 8j)

## Data Availability

RNA-seq data analysis was performed with fastp (https://github.com/OpenGene/fastp) and HISAT2 version 2.0.5 (https://daehwankimlab.github.io/hisat2/). For differential expression and functional enrichment analysis, DESeq2 version 1.16.1 and clusterProfiler version 3.4.4 were used: http://bioconductor.org/packages/3.15/bioc/. Proteomic raw data were processed using Proteome Discoverer version 2.4 (https://www.thermofisher.com/order/catalog/product/OPTON-30810#/OPTON-30810), NAguildeR (https://www.omicsolution.com/wukong/NAguideR/), limma (https://www.omicsolution.com/wkomics/passwd/hytestlimma/), STRING version 11.5 (https://cn.string-db.org), and cystoscope version 3.9.0 (https://github.com/cytoscape/cytoscape/releases/3.9.0/).
